# *Cinnamomum verum* J. Presl Bark Contains High Contents of Nicotinamide Mononucleotide

**DOI:** 10.3390/molecules27207054

**Published:** 2022-10-19

**Authors:** Jing Yan, Takumi Sakamoto, Ariful Islam, Yashuang Ping, Soho Oyama, Hiroyuki Fuchino, Hitomi Kawakami, Kayo Yoshimatsu, Tomoaki Kahyo, Mitsutoshi Setou

**Affiliations:** 1 Department of Cellular & Molecular Anatomy, Hamamatsu University School of Medicine, Hamamatsu 431-3192, Shizuoka, Japan; 2 Preppers Co., Ltd., 141 Innovative Medical Collaboration Building, 1-20-1 Handayama, Higashi-ku, Hamamatsu 431-3192, Shizuoka, Japan; 3 Tsukuba Division, Research Center for Medicinal Plant Resources, National Institutes of Biomedical Innovation, Health and Nutrition (NIBIOHN), 1-2 Hachimandai, Tsukuba 305-0843, Ibaraki, Japan; 4 International Mass Imaging Center, Hamamatsu University School of Medicine, 1-20-1 Handayama, Higashi-ku, Hamamatsu 431-3192, Shizuoka, Japan; 5 Department of Systems Molecular Anatomy, Institute for Medical Photonics Research, Preeminent Medical Photonics, Education & Research Center, 1-20-1 Handayama, Higashi-ku, Hamamatsu 431-3192, Shizuoka, Japan

**Keywords:** anti-aging, nicotinamide mononucleotide (NMN), *Cinnamomum verum* J. Presl (*C. verum*), UPLC-MS/MS, Plant Extract Library (PEL), traditional medicine

## Abstract

The global population is aging, and intervention strategies for anti-aging and the prevention of aging-related diseases have become a topic actively explored today. Nicotinamide adenine dinucleotide (NAD^+^) is an important molecule in the metabolic process, and its content in tissues and cells decreases with age. The supplementation of nicotinamide mononucleotide (NMN), an important intermediate and precursor of NAD^+^, has increased NAD^+^ levels, and its safety has been demonstrated in rodents and human studies. However, the high content of NMN in natural plants has not been fully explored as herbal medicines for drug development. Here, we identified that the leaf of *Cinnamomum verum* J. Presl (*C. verum*) was the highest NMN content among the Plant Extract Library (PEL) with food experience, using ultra-performance liquid chromatography–tandem mass spectrometry (UPLC-MS/MS). To validate this result, the extraction and quantitative analysis of bark, leaf, root, and stem of fresh *C. verum* was conducted. The results revealed that the bark had the highest NMN content in *C. verum* (0.471 mg/100 g). Our study shed light on the prospects of developing natural plants in the context of NMN as drugs for anti-aging and prevention of aging-related diseases. The future should focus on the development and application of *C. verum* pharmaceutical formulations.

## 1. Introduction

In recent years, the global population has been rapidly aging, especially in developed and developing countries where life expectancy is extending. With population aging comes an increase in the prevalence of age-related neurodegenerative diseases, such as cardiovascular disease, diabetes, cancer, and cognitive impairment due to Alzheimer’s disease [1,2]. In other words, the healthy life expectancy is not keeping up with the longevity of the human population [3,4]. In this context, the United Nations has launched the Decade of Healthy Ageing initiative to respond to population aging. Current interventions for aging include public health measures to reduce the risk of aging-related diseases, lifestyle modifications, and drug treatments targeting specific cells and molecules; however, lifestyle modifications and preventive drug treatments are more achievable in terms of compliance [5].

The supplementation with NAD^+^ precursors nicotinamide riboside (NR), nicotinamide mononucleotide (NMN), nicotinic acid (NA), and nicotinamide (NAM) to ameliorate age-related pathophysiology and disease conditions have been extensively studied in recent years [6,7]. NAD^+^ is an important coenzyme for redox reactions and central to energy metabolism [8]. It can affect many critical cellular functions [9], including metabolic pathways, DNA repair, chromatin remodeling, cellular senescence, immune cell function, and is vital for maintaining tissue and metabolic homeostasis and healthy aging [10]. With age, over-activation of NAD-consuming enzymes such as poly (ADP-ribose) polymerase (PARP), sterile alpha, TIR-containing pattern 1 (SARM1), and CD38 leads to NAD^+^ depletion [11]. Intracellular NAD^+^ synthesis is achieved mainly through the NAM salvage pathway mediated by NAMPT and NMN adenylyltransferases (NMNAT1-3) in sequence [12]; downregulation of NAMPT causes the reduction in NAD^+^ synthesis with aging [13]. In mammalian cells, except for neurons, NAD^+^ is not directly absorbed by cells, and therefore, cannot be replenished [14]. Recent studies have shown that supplementation with NMN (a precursor of NAD^+^) is effective in increasing NAD^+^ biosynthesis in various peripheral tissues and the brain. It has excellent potential for the treatment and improvement of vascular cognitive impairment [15,16], type 2 diabetes [17], age-related decline in fertility [18], physiological decline [19], and neurodegenerative pathologies such as Alzheimer’s disease [20,21]. 

Traditional medicines using natural products such as Traditional Chinese Medicine (TCM), Kampo, Traditional Korean Medicine (TKM), etc., are an essential part of modern medicine [22]. Natural products of plant origin have abundant compounds and activities, a wide range of action, and a low rate of toxic effects [23]. Their synergistic effects between compounds and multi-target therapeutic effects are irreplaceable by chemically synthesized drugs [24]. However, the current research on anti-aging properties of traditional drugs has not yet been fully explored. In recent years, dietary supplements containing natural NMN have been widely commercialized, but they are only sold as functional foods rather than strictly regulated therapeutic drugs [25]. Natural products have been found to contain many anti-aging active compounds [26], such as Berberine in Coptis chinensis, Coptis japonica, Coptis rhizome [27], and resveratrol in Reynoutria japonica [28,29]. While the exploration of NMN in natural products is not completely clear. We use the known anti-aging potential of NMN, combined with the advantages of medicinal plants over chemically synthesized drugs, it is a promising strategy to explore plants with high NMN content from natural products as future anti-aging medicinal plant development.

Ultra-performance liquid chromatography–tandem mass spectrometry (UPLC-MS/MS) has been widely used for the separation and rapid identification of compounds in natural products [30] due to its higher sensitivity, selectivity and stability. Recently, the Plant Extract Library (PEL) has been used for various screening and development [31]. We used UPLC-MS/MS analysis technology and high-throughput methods to screen for NMN in the PEL. We also used a simple plant extraction method to extract and measure the fresh plants with high NMN content screened accordingly, i.e., *Cinnamomum verum* J. Presl (*C. verum*, fam. Lauraceae). *C. verum* has a long history of use as a food, spice, and traditional medicine. Modern analytical techniques have detected and identified parts of the compounds in *C. verum* extracts [32]. For example, (E)-cinnamaldehyde, eugenol, (E)-caryophyllene, (E)-cinnamyl acetate, and α-humulene as the five compounds were major identified using gas chromatography–mass spectrometry [33]; LC-MS analysis of *C. verum* bark extracts identified trans-cinnamaldehyde and cinnamic acid as well as A- and B-type proanthocyanidins [34]; Gas chromatography-flame ionization detector, GC-MS analysis, and chemical synthesis identified various compounds in the essential oil of *C. verum* [35]. In our study, we demonstrated the high content of NMN in *C. verum* bark, which gives additional information on the compounds in *C. verum* and provides new ideas and evidence for anti-aging traditional medicine of NMN.

## 2. Results

### 2.1. Establishment of the Appropriate UPLC-MS/MS Method to Detect NMN 

**Figure 1:**
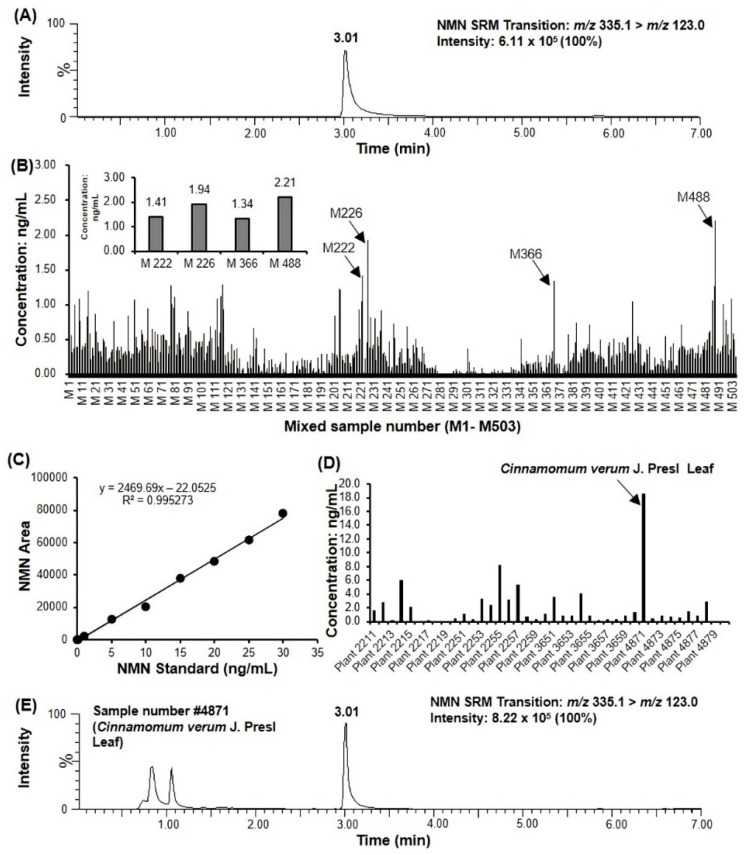
*Cinnamomum verum* J. Presl (*C. verum*) leaf had the highest nicotinamide mononucleotide (NMN) content in the Plant Extract Library (PEL). (**A**) The chromatogram of the NMN standard was measured by UPLC-MS/MS. (**B**) Screening of NMN in the PEL (5022 samples) by the mixing pool method; 10 and 2 samples were mixed into one pool in mixing pool numbers M1 to M502 and M503, respectively. The insert shows the top four mixing pools with the highest NMN content. (**C**) Standard curve of NMN for quantitative analysis by UPLC-MS/MS. The standard curve was generated using linear regression. (**D**) Forty samples from the top four mixing pools with the highest NMN concentration (**B**) were measured individually and quantified using the NMN standard curve. The sample #4871 (*C. verum* leaf) had the highest NMN content of about 20 ng/mL. (**E**) The chromatogram of NMN in sample #4871.

In the first step, the chromatographic method for the detection and quantification of NMN was established. During the optimization of the separation conditions, the Intrada Amino Acid column (3 μm, 100 × 3.0 mm, Imtakt, Kyoto, Japan) showed the best separation and peak shape. Optimal mobile phase and gradient conditions were sought in order to obtain the best separation of standards. Acetonitrile with 0.3% Formic acid and Acetonitrile/water (20:80, *v*/*v*) with 100 mM ammonium formate as well as the gradient described in the method 4.5 were determined as the final chromatographic conditions. Then, the mass spectrometry method was optimized in positive ion mode before measuring the plant extract samples using the NMN standard. After the collision of the [M+H]^+^ precursor ion at atmospheric pressure, the highest intensity fragment ion was selected as the product ion, which was not detected in the blank solvent. The transition from *m*/*z* 335.1 to 123.0 with high intensity and minimal interference was established in selected reaction monitoring (SRM) mode for the selected ion. The collision intensity and voltage were also optimized to obtain the optimal signal intensity. Finally, NMN was successfully isolated from the NMN standard (Figure 1A).

### 2.2. Screening of the Plant Extract Library to Acquire the Plant Samples with the Highest NMN Content 

In the present study, to rapidly detect as many as 5022 samples in the PEL, we used a high-throughput mixing pool method to pretreat the samples. The NMN content among a total of 503 mixing pools was quantitatively measured and analyzed by established UPLC-MS/MS after sample preparation (Figure 1B). The correlation coefficients for each calibration curve in the measurements for the M1-M503 samples are summarized in Table S1. Then, we selected the top four pools with the highest NMN content, M222, M226, M366 and M488 (Figure 1B), and the total of 40 samples from those pools were measured individually. The linear equation y = 2469.69x − 22.0525 (R² = 0.9953) was obtained using the concentration of NMN standard as x and the detected NMN peak area as y. The linearity was well in the concentration range of 0.1–30 ng/mL (Figure 1C). The results showed that the highest NMN content (0.465 mg/100 g) was obtained from Plant number 4871 (Figure 1D,E), which was *Cinnamomum verum* J. Presl (*C. verum*, fam. Lauraceae) leaf sample grown in the greenhouse in Kanto, Japan, and harvested on 8 May 2013.

### 2.3. Detection of NMN Content in Cinnamomum verum J. Presl

**Figure 2:**
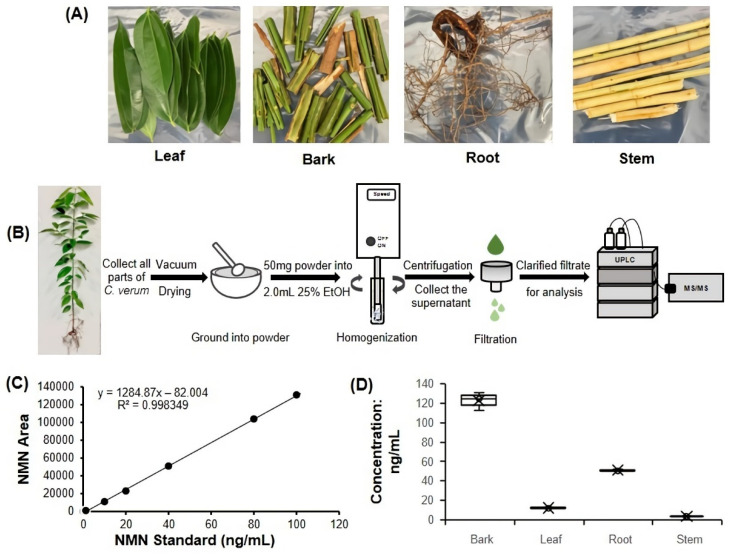
NMN detection in *Cinnamomum verum* J. Presl. (**A**) Pictures of different parts of *C. verum* were used for our measurements. (**B**) Sample preparation process. (**C**) Plot of the NMN standard curve. Standard curve of NMN in UPLC-MS/MS quantitative analysis; the standard curve was generated using linear regression. (**D**) UPLC-MS/MS measurement results after sample preparation with dry powder of each part of *C. verum* (*n* = 3 per group, relative standard deviation (RSD) of bark, leaf, root and stem were 7.93%, 6.14%, 2.32%, and 2.70%, respectively).

To confirm the high content of NMN in *C. verum*, we next conducted the extraction of bark, leaf, root and stem parts of the fresh *C. verum* tree (Figure 2A) by the simple and rapid plant extraction method (Figure 2B). The linear equation y = 1284.87x − 82.004 (R² = 0.9983) was also measured and calculated, with good linearity in the range of 0.5–100 ng/mL (Figure 2C). The results proved that bark contains the highest NMN content among different parts of cinnamon plant (Figure 2D). *Cinnamomum loureiroi* (*C. loureiroi,* fam. Lauraceae) is the same genus as *C. verum* and has been used as an economically important species similarly to *C. verum*. Cabbage (*Brassica oleracea* var. *capitata,* fam. Brassicaceae) has been reported in the reference study with high NMN content. Therefore, we compared the *C. verum* samples, *C. loureiroi* bark and Cabbage leaf prepared with the same method (Table 1). *C. verum* bark was 0.471 mg/100 g while *C. loureiroi* bark and Cabbage leaf were 0.133 and 1.207 mg/100 g, respectively.

## 3. Discussion

The purpose of this study is to explore plants with high NMN content for anti-aging drug development. We screened 5022 plant extract samples with food experience from 27 countries and regions around the world, covering 148 plant families. In our study, to screen PEL samples quickly and efficiently, we applied a high-throughput mixed sample pool method for sample preparation combined with UPLC-MS/MS technology to develop a 7 min rapid measurement method. We also conducted quantitative analysis of extract from the fresh plants. In this step, we utilized a simple plant sample extraction process and eliminated the effect of enzymes in the plant samples on NMN.

To verify the screening results of the PEL, we acquired commercially available *C. verum* trees for the measurements. The extraction method of the samples in the PEL provided by the Tsukuba Division, Research Center for Medicinal Plant Resource, National Institutes of Biomedical Innovation, Health and Nutrition (NIBIOHN) was the reflux method, and due to our limited experimental conditions, the homogenization method was used. As the different extraction methods, the bark of *C. loureiroi* Cabbage leaves were extracted by homogenization and analyzed for relative comparison with fresh *C. verum* samples. *C. verum* translation “true cinnamon”, also known as Ceylon cinnamon, originated from Sri Lanka; *C. loureiroi* is also known as Vietnamese or Saigon cinnamon. They belong to the common commercial species of the *Cinnamomum* spp. and were usually used as food, spices, and traditional medicines. Our results show that fresh *C. verum* bark contains higher NMN than *C. loureiroi* bark (Table 1). In the available studies, *C. verum* and *C. loureiroi* bark contents of the compounds were varied except for the main content of cinnamaldehyde [36]. The measured NMN content in Cabbage leaves was approximately 1.3-fold of the highest value in reference [19]. The extraction method in the reference was not described and may differ from ours; however, the results are approximate. The NMN content in fresh *C. verum* leaf was 0.052 mg/100 g, which was 11.2% of that in *C. verum* leaves in the PEL and about 11.0% of that in fresh *C. verum* bark. In fact, for different plant species, extraction methods, storage conditions, age, harvest time, geographic cultivation area, etc., the compound fractions of the plants will vary [37,38]. Therefore, the differences between the NMN content of *C. verum* bark and *C. loureiroi* bark, *C. verum* leaves from PEL and fresh *C. verum* leaves can be attributed to the above factors. In the preparation of natural plants as traditional medicine, the acquisition of plant samples and extraction methods should be the focus of attention. Nevertheless, our results demonstrate that *C. verum* has a high content of NMN, and the bark was the highest NMN containing parts of in *C. verum* under the same conditions. In the future, we need more studies to elucidate the range of NMN content in *C. verum*.

During further measurements on *C. verum* bark, we found that the level of NMN in the extract decreased with time (Figure S1). We considered that the enzymes in the plant degraded NMN. In order to verify and eliminate this effect, the plants were heat treated. Before performing this step, NMN standard were first heat treated under different conditions to ensure that NMN was unaffected (Figure S2). The fresh *C. verum* bark was selected for heat treatment at 100 °C for 3 min, and NMN level was stabilized within 24 h at 0.703 mg/100 g with relative standard deviation (RSD) of 3.71% (Figure S1). The natural plant contains abundant compounds, but the extraction process will produce unknown reactions; thus, the extraction method significantly affects the extraction efficiency. While in this experiment, the NMN content was measured as stable after the heat treatment.

*C. verum* has been shown to be effective as an adjuvant in controlling diabetes [39], reducing blood glucose and lipid levels in patients with type 2 diabetes [40], improving the antioxidant status of female reproductive dysfunction-related disorders such as polycystic ovary syndrome [41,42], in cancer [43], and in neurodegenerative diseases such as Alzheimer’s disease and Parkinson’s disease with some therapeutic effects [44,45]. On the other hand, NMN has been shown to have great potential in treating type 2 diabetes [17], improving female reproductive aging caused by reduced fertility [18], Alzheimer’s disease and other aging-related diseases and neurodegeneration [20,21]. These findings led us to assume that some anti-aging effects could be attributed to the compound NMN or a synergistic effect between NMN and other compounds. Regarding the high content of NMN in *C. verum*, it can be suggested to use *C. verum* as a research direction for drug development and treatment for age-related diseases. 

In the aspect of safe cinnamon edibility, coumarin is known to be the main toxic substance present in cinnamon. According to the European Food Safety Authority, the human daily tolerance for coumarin is 0.1 mg/kg [46]. Among the several commercially available cinnamon species, *C. verum* contains the lowest content of coumarin: 0.017 mg/g [47], indicating the edible safety of *C. verum* and providing strong support for its use as a dietary supplement and drug development.

In conclusion, our study found that *C. verum* contains high NMN, especially the highest NMN content in *C. verum* bark, which gives additional information of the compounds in *C. verum*. This will provide support and ideas for further research on anti-aging and the development of pharmaceuticals for the treatment of aging-related diseases.

## 4. Materials and Methods

### 4.1. Reagents and Chemicals

The standard β-nicotinamide mononucleotide (β-NMN) was purchased from Tokyo Chemical Industry Co. (Tokyo, Japan). The 5022 samples of the PEL were provided and the details of the samples, including the location and date of collection of the plant, scientific name, extraction amount, and the part of the extraction specimen were recorded and managed by the Tsukuba Division, Research Center for Medicinal Plant Resources, National Institutes of Biomedical Innovation, Health and Nutrition (NIBIOHN). The information regarding the samples was recorded and managed with unique numbers [31]. *C. verum* aged about 12–18 months was obtained from a gardening company (Chubu Building Hozen, Shizuoka, Japan). Standard and PEL were transported frozen and stored at −20 °C until use. LC/MS-grade of ultra-pure water, acetonitrile (ACN), methanol, ethanol (99.5), and formic acid (99%) were obtained from FUJIFILM Wako Pure Chemical Corporation, Ltd. (Osaka, Japan). Additionally, special grade reagent dimethyl sulfoxide (DMSO) was obtained from Wako Pure Chemical Industries, Ltd. (Osaka, Japan). Ammonium formate solution (1 mol/L) was purchased from Kanto Chemical Co., INC. (Tokyo, Japan).

### 4.2. Standard Preparation

For the preparation of standards, NMN standards were weighed using an analytical balance (AUY220, SHIMADZU, Kyoto, Japan). The NMN standard used for PEL screening was dissolved in 10% DMSO solvent and serially diluted to 30, 25, 20, 15, 10, 5, 1, and 0.1 ng/mL control solution. For the measurement of *C. verum*, *C. loureiroi*, and Cabbage plant extract samples, NMN standard was dissolved in 25% ethanol, serially diluted to 100, 80, 40, 20, 10, 1, and 0.5 ng/mL control solutions. These standards were used to generate calibration curves to measure NMN concentrations in PEL and plant extracts.

### 4.3. Plant Extract Library Preparation for UPLC-MS/MS Screening 

The obtained PEL (5022 samples) was dissolved in DMSO at a concentration of 40 mg/mL and packed in 96-well plates. The samples were mixed with Microplate mixer (MPX-96, scinics, Tokyo, Japan) at room temperature to melt and mix well. Pretreatment was performed using the high-throughput mixing pool method: 10 µL of each plant extract sample were taken and pre-mixed; every 10 and 2 samples were mixed into one pool in mixing pool numbers M 1 to M 502, and M 503, respectively. After mixing well, 50 µL (M 1 to M 502) and 10 µL (M503) of the mixed plant extract were added to 450 µL and 90 µL of pure water, respectively, and vortexed; the mixture and NMN standard was subsequently centrifuged at 13,000 rpm for 10 min at 4 °C (Eppendorf Centrifuge 5424 R). Then, 50 µL of supernatant were filtered using a 0.20 µm filter (SLLGH04NL, Merck Millipore, Tokyo, Japan). Finally, the filtered clarified sample was transferred to a glass vial (L-2ML-9-V1002, LE Technologies, Saitama, Japan) with micro inserts (98024, Systech, Tokyo, Japan) and stored at −20 °C until UPLC-MS/MS analysis was performed.

### 4.4. Cinnamomum verum J. Presl Extract Preparation for UPLC-MS/MS 

Fresh *C. verum* was washed with running water and then rinsed with deionized water. The bark, leaves, stems, and roots were separated and then vacuum-dried (Figure 2B). Drying was conducted until the weight of sample stopped decreasing. The harder material’s roots, stems, and bark were first cut manually, and then the plant material was immediately ground into powder [48]. Approximately 50 mg was weighed (parallel three of each plant part sample), added to 2 mL of 25% ethanol (40 µL/mg), and extracted by homogenization (HK-1, AS ONE, Osaka, Japan) on the ice at 9000 rpm for 2 min. The homogenized plant sample extracts were subsequently centrifuged at 13,000 rpm for 15 min at 4 °C; 50 µL of supernatant was filtered using a 0.20 µm filter. In a subsequent heat treatment optimization step, filtered samples were heat-treated with a heat block (BI-515, ASTE, Fukuoka, Japan) at 100 °C for 3 min. Finally, the cleared sample was transferred to a glass vial with micro inserts and stored at −20 °C until UPLC-MS/MS analysis was performed.

### 4.5. UPLC-MS/MS Method

PEL samples and *C. verum* extract were analyzed using an Acquity Ultra-Performance Liquid Chromatography System (H-Class PLUS, Waters, Milford, MA, USA) coupled with a triple quadrupole mass spectrometer (Xevo-TQ-XS, Waters). Chromatographic separation was performed using an Intrada Amino Acid column (3 μm, 3.0 × 100 mm, Imtakt), which was maintained at 40 °C during analysis. Mobile phase A for PEL screening and plant extract samples analysis consisted of 0.3% formic acid in ACN and mobile phase B was consisted of acetonitrile/100 mM ammonium formate (20:80, *v*/*v*) with an elution flow rate of 0.6 mL/min. The gradient elution procedure used for PEL screening and plant extracts were set up as follows: 0–0.5 min, 20% B; 0.5–2.5 min, 20%–100% B; 2.5–4.5 min, 100% B; 4.5–4.6 min, 100%–20% B; 4.6–7.0 min, 20% B. The total elution time was 7 min. The autosampler temperature was maintained at 10 °C with an aliquot of 5 μL of the prepared sample injected into the system. The mass spectrometer was equipped with an electrospray ionization (ESI) source, and samples were analyzed in positive mode. The SRM was optimized using β-NMN standards. [M+H]^+^ was chosen as the precursor ion. The transition from *m*/*z* 335.1 to 123.0 using collision energy (CE) 10.0 V was used for the mass number of protonated NMN ions and production. The capillary voltage was set at 4.0 kv, the cone voltage at 22 V, and the source temperature at 150 °C. The desolvation temperature was set at 500 °C with a desolvation gas flow of 600 L/h. The gas flow of the cone and nebulizer was set at 150 L/h and 7.0 Bar, respectively. 

### 4.6. Measurement from the Fresh Plant

The minimum limit of detection of NMN was determined as 0.1 ng/mL with the signal-to-noise ratio (S/N) >3, and the minimum limit of quantification of NMN was determined as 0.2 ng/mL with the target peak S/N > 10 (Figure S3). The NMN standard at 100 ng/mL was repeatedly injected 6 times and the peak area was recorded with an RSD of 7.896%, indicating that the precision of the instrument was well (Figure S4). *C. verum* bark powder was weighed in parallel in 6 equal parts, and the sample was prepared according to the method described in 4.4. After measuring the sample, the peak area was recorded, and the RSD of NMN content in the sample was calculated: 4.962%, which indicated that the plant extract method was reproducible (Figure S5). Three parallel weights of *C. verum* powder were prepared according to the method described in 4.4, and the samples were measured at 0, 12, and 24 h. The peak areas of NMN were measured, and the RSD of NMN content in the samples was calculated as 3.17%, indicating that the method had satisfactory stability at 24 h (Figure S1).

### 4.7. UPLC-MS/MS Data Analysis

The UPLC-MS/MS data acquisition, analysis, and control of the system were performed by MassLynx (Waters, Milford, MA, USA; version 4.1) software. The signal-to-noise ratio is calculated based on the maximum signal height above the mean noise divided by the root-mean-square deviation of the mean noise.

## Figures and Tables

**Table 1:** NMN content in different plants.

Plant Name (Source or English Name)	Plant Part Used	Extraction Solution	mg/100 g ^1^
*C. verum* (PEL, Ceylon cinnamon)	Leaf	Methanol	0.465
*C. verum* (Ceylon cinnamon)	Leaf	25% Ethanol	0.052
*C. verum* (Ceylon cinnamon)	Bark	25% Ethanol	0.471
*C. verum* (Ceylon cinnamon)	Root	25% Ethanol	0.201
*C. verum* (Ceylon cinnamon)	Stem	25% Ethanol	0.016
*C. Loureiroi* (Vietnamese cinnamon)	Bark	25% Ethanol	0.133
*B. oleracea* var. *capitata* (Cabbage)	Leaf	25% Ethanol	1.207
*B. oleracea* var. *capitata* (Cabbage) [19]	-	-	0.0–0.90

NMN content in plants was measured by UPLC-MS/MS in several experiments. *C. verum* (PEL) leaves were screened in PEL as shown in Section 2.2. The measured mean values for *C. verum* bark, leaves, roots, and stems are shown in Table 1, with RSD shown as 7.93%, 6.14%, 2.32%, and 2.70%, respectively. *n* = 3 per group. *C. Loureiroi* bark and *B. oleracea* var. *capitata* leaf RSD were: 11.04%. and 2.34%, respectively. *n* = 2 per group. ^1^ The weighing state of the reference Cabbage: no information, the weighing state of other plants: dry powder.

## Data Availability

Data are contained within the article or Supplementary Material.

## Supplementary Materials

The following supporting information can be downloaded at: https://www.mdpi.com/article/10.3390/molecules27207054/s1, Figure S1: Comparison of NMN content of heat treatment and control (non-heat treatment) in *C. verum* bark; Figure S2: Statistical analyses of heat treated under different conditions by UPLC-MS/MS; Figure S3: The minimum limit of detection and the minimum limit of quantification of NMN by UPLC-MS/MS; Figure S4: The precision test of the UPLC-MS/MS instrument; Figure S5: Plant extraction method reproduction test; Table S1: List of correlation coefficients of calibration curves for measurement of M1-M503 samples.

## Sample Availability

Samples of the compounds are not available from the authors.
