# Supplementary material for: Cinnamomum verum J. Presl Bark Contains High Contents of Nicotinamide Mononucleotide
Source: Molecules. 2022 Oct 19;27(20):7054. doi: 10.3390/molecules27207054 (PMC9612253; doi:10.3390/molecules27207054)
Supplement: Supplementary file 1 [file molecules-27-07054-s001.zip › 220929_Supplementary Materials.pdf]

Supplementary Materials

Article

Cinnamomum verum J. Presl Bark Contains High Contents of Nicotinamide Mononucleotide

Jing Yan [1], Takumi Sakamoto [1,2], Ariful Islam [1,2], Yashuang Ping [1], Soho Oyama [1], Hiroyuki Fuchino [3], Hitomi Kawakami [3], Kayo Yoshimatsu [3], Tomoaki Kahyo [1,4] and Mitsutoshi Setou [1,4,5,*]

[1] Department of Cellular & Molecular Anatomy, Hamamatsu University School of Medicine, Hamamatsu, Shizuoka 431-3192, Japan
[2] Preppers Co. Ltd., 141 Innovative Medical Collaboration Building, 1-20-1 Handayama, Higashi-ku, Hamamatsu, Shizuoka 431-3192, Japan
[3] Tsukuba Division, Research Center for Medicinal Plant Resources, National Institutes of Biomedical Innovation, Health and Nutrition (NIBIOHN), 1-2 Hachimandai, Tsukuba, Ibaraki 305-0843, Japan
[4] International Mass Imaging Center, Hamamatsu University School of Medicine, 1-20-1, Handayama, Higashi-Ku, Hamamatsu, Shizuoka 431-3192, Japan
[5] Department of Systems Molecular Anatomy, Institute for Medical Photonics Research, Preeminent Medical Photonics, Education & Research Center, 1-20-1 Handayama, Higashi-Ku, Hamamatsu, Shizuoka 431-3192, Japan
* Correspondence: setou@hama-med.ac.jp; Tel.: +81-053-435-2086; Fax: +81-053-435-2468

Figure S1

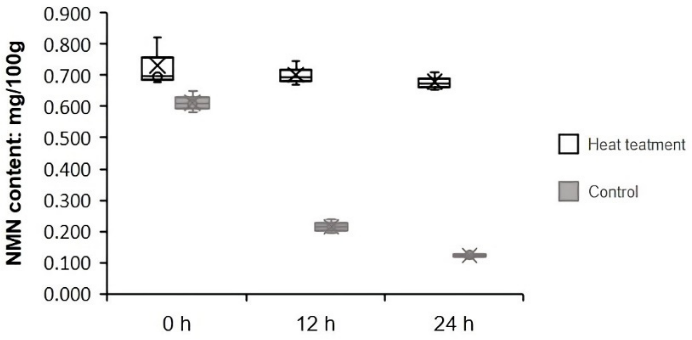

Figure S1: Comparison of NMN content of heat treatment and control (non-heat treatment) in *C. verum* bark. Heat treatment was performed after the extraction step of Figure 2B. The NMN content in *C. verum* bark with heat treatment was 0.703 mg/100g with RSD: 3.71% in 24 hours; The NMN content of control degraded with time (n=3 per group).

Figure S2

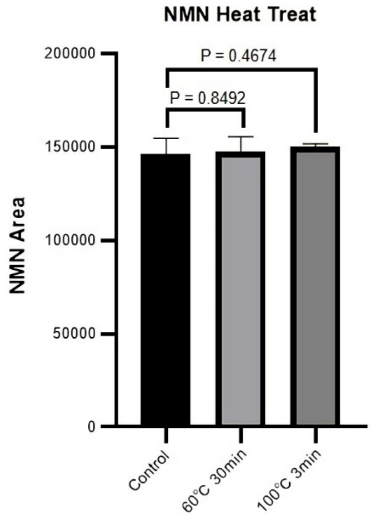

Figure S2: Statistical analyses of heat treated under different conditions by UPLC-MS/MS. Heat treatment using NMN standard 100 ng/mL under different conditions (control: non-heat treatment, heat treatment at 60°C for 30 min and heat treatment at 100°C for 3 min). Unpaired T-test (n=3 per group), all data represent as mean ± SD.

Figure S3

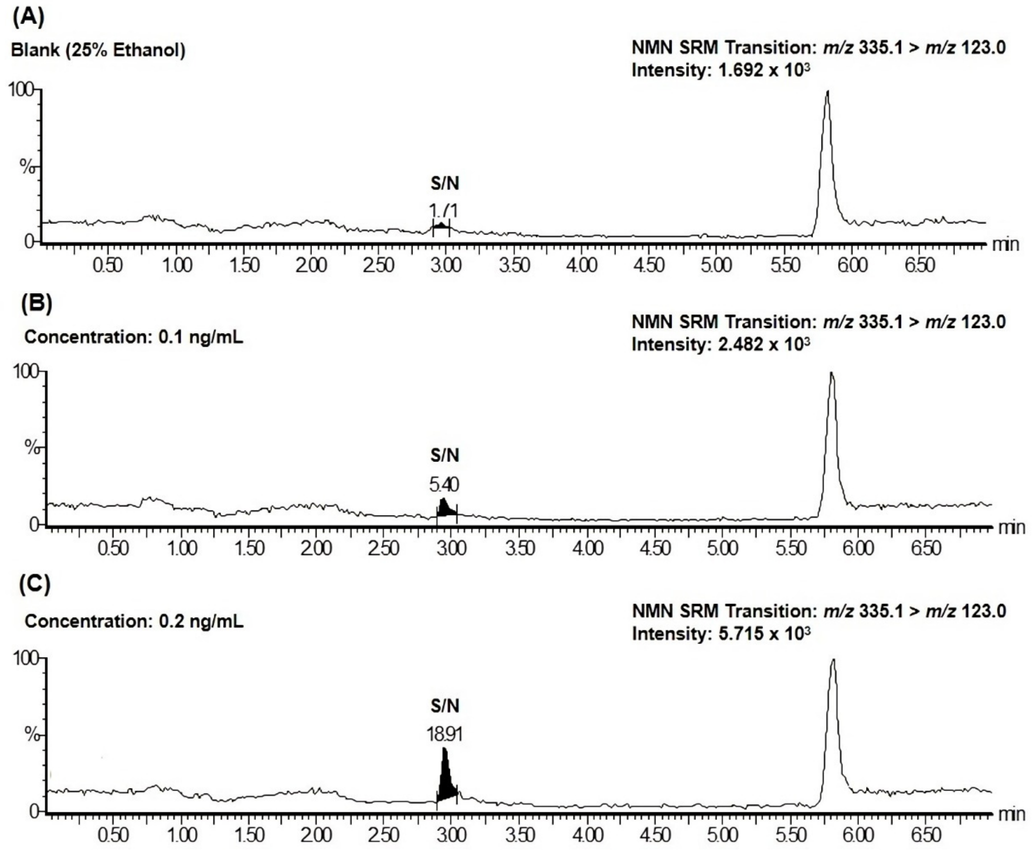

Figure S3: The minimum limit of detection and the minimum limit of quantification of NMN by UPLC-MS/MS. (A) NMN was detected in Blank (25% Ethanol) with the signal-to-noise ratio (S/N) of 1.71. (B) The minimum limit of detection (0.1 ng/mL) of NMN with the S/N of 5.40. (C) The minimum limit of quantification (0.2 ng/mL) of NMN with the S/N of 18.91.

Figure S4

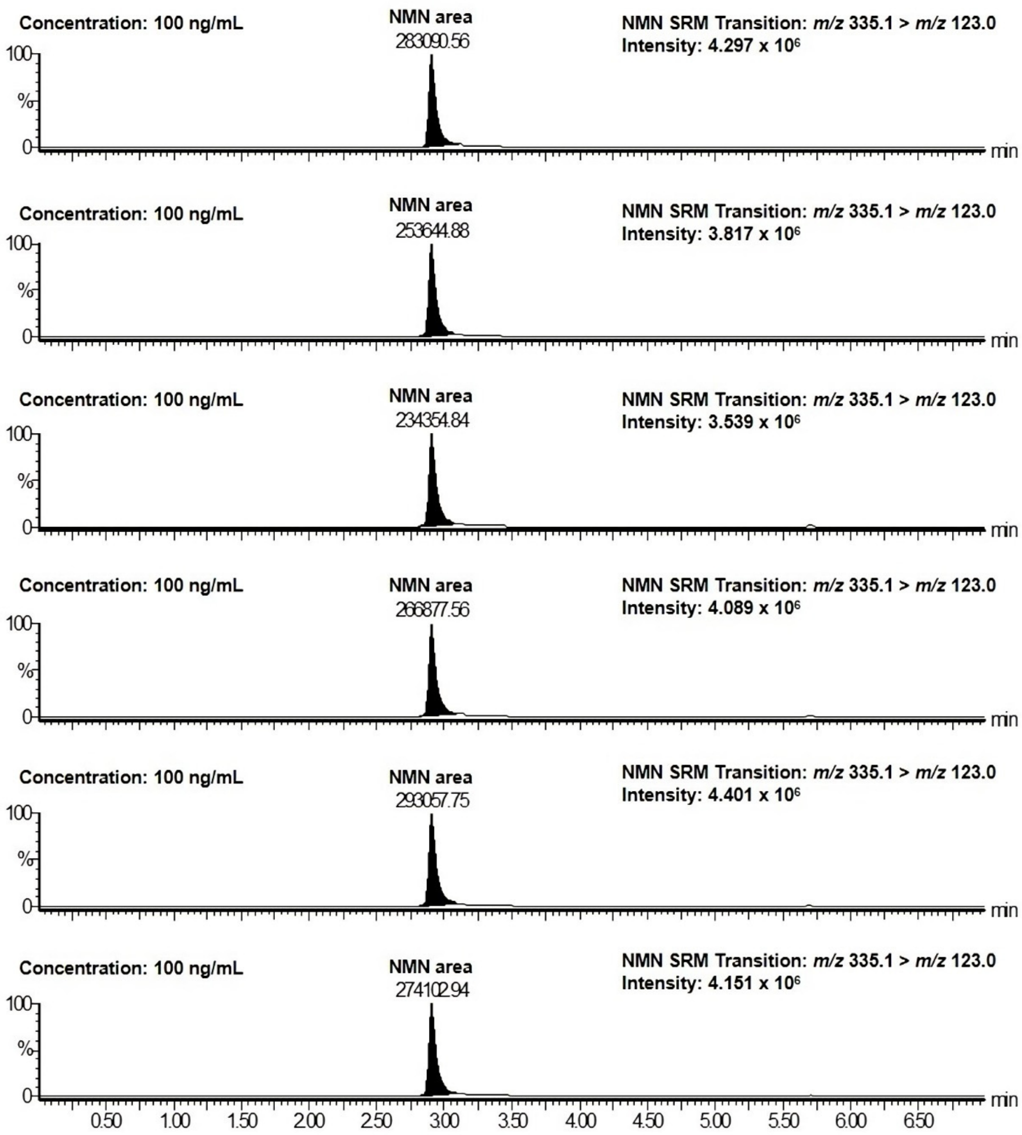

Figure S4: The precision test of the UPLC-MS/MS instrument. The NMN standard at 100 ng/mL in 25% Ethanol was repeatedly injected 6 times, and the peak area was recorded with an RSD of 7.896%.

Figure S5

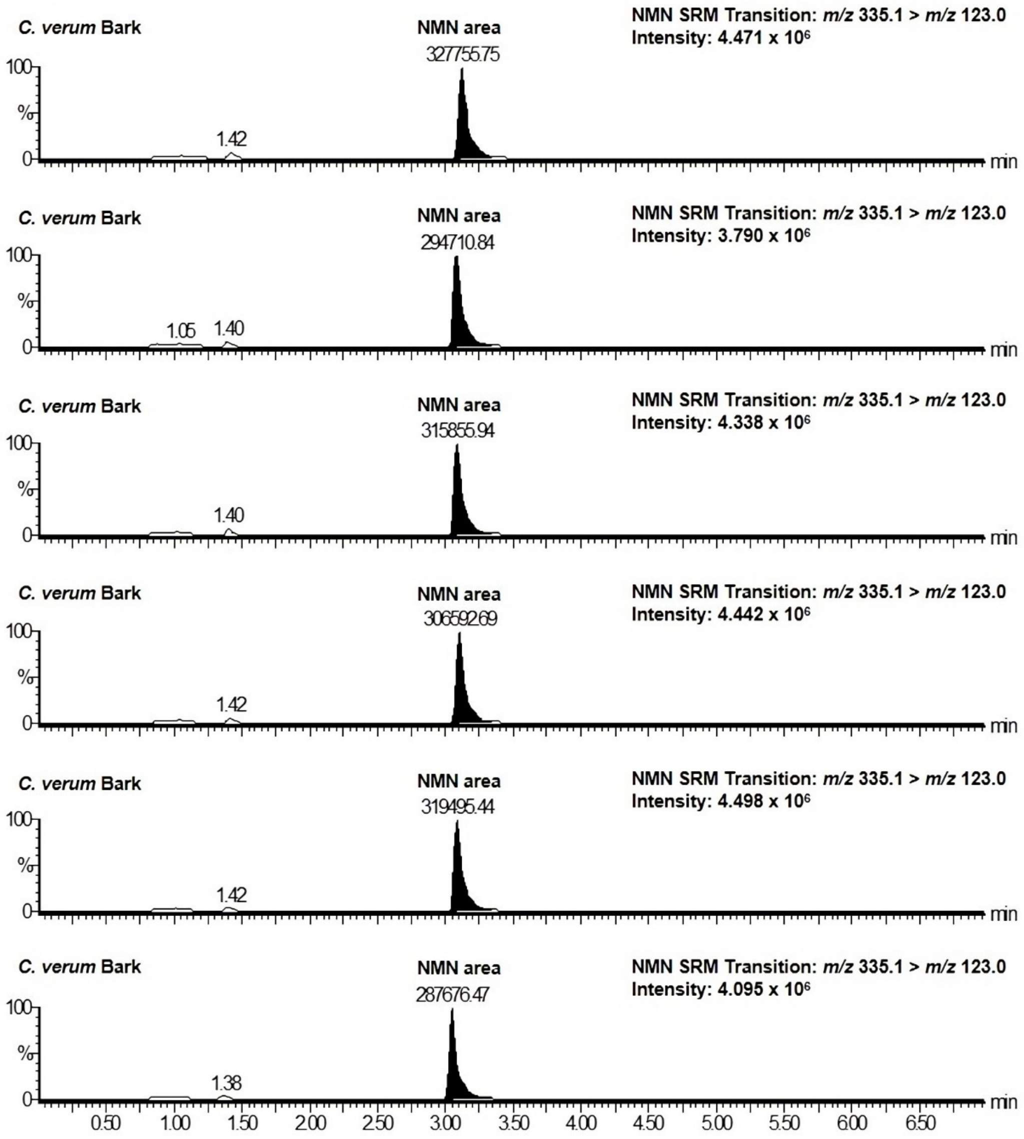

Figure S5: Plant extraction method reproduction test. *C. verum* bark powder was weighed in parallel in 6 equal parts, and the sample was prepared according to the method 4.4. After measuring the sample, the peak area was recorded, and the RSD of NMN content in the sample was calculated: 4.962%.

Table S1: List of correlation coefficients of calibration curves for measurement of M1-M503 samples.

Plan Mixing pool No.	Calibration curve	R^2
M1-M117	y = 1873.3x - 320.74	0.9990
M118-M197	y = 2128.9x + 125.36	0.9996
M198-M217	y = 2165.2x - 111.16	0.9842
M218-M253	y = 2668.1x - 37.015	0.9998
M254-M317	y = 3128.3x + 26.532	0.9989
M318-M376	y = 3108.1x - 30.552	0.9997
M377-M417	y = 2638.9x - 236.89	0.9889
M418-M459	y = 2459.6x - 147.42	0.9989
M460-M503	y = 2549.3x - 555.78	0.9967
