# Supplementary figures and images for: Cinnamomum verum J. Presl Bark Contains High Contents of Nicotinamide Mononucleotide
Source: Molecules. 2022 Oct 19;27(20):7054. doi: 10.3390/molecules27207054 (PMC9612253; doi:10.3390/molecules27207054)

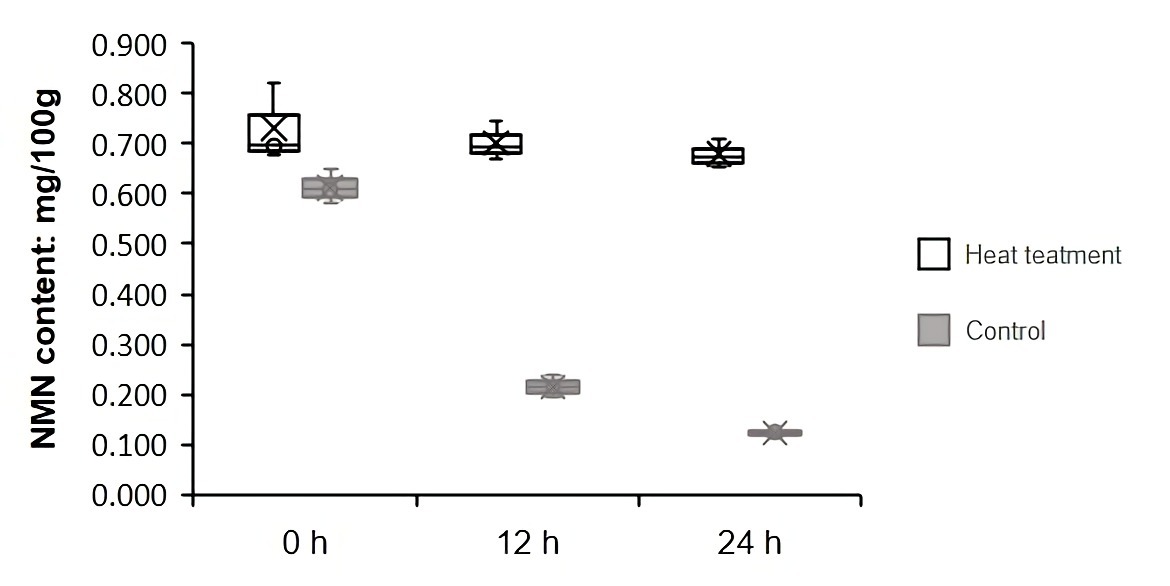

Supplement: Supplementary file 1 [file molecules-27-07054-s001.zip › S1 (1).jpg]

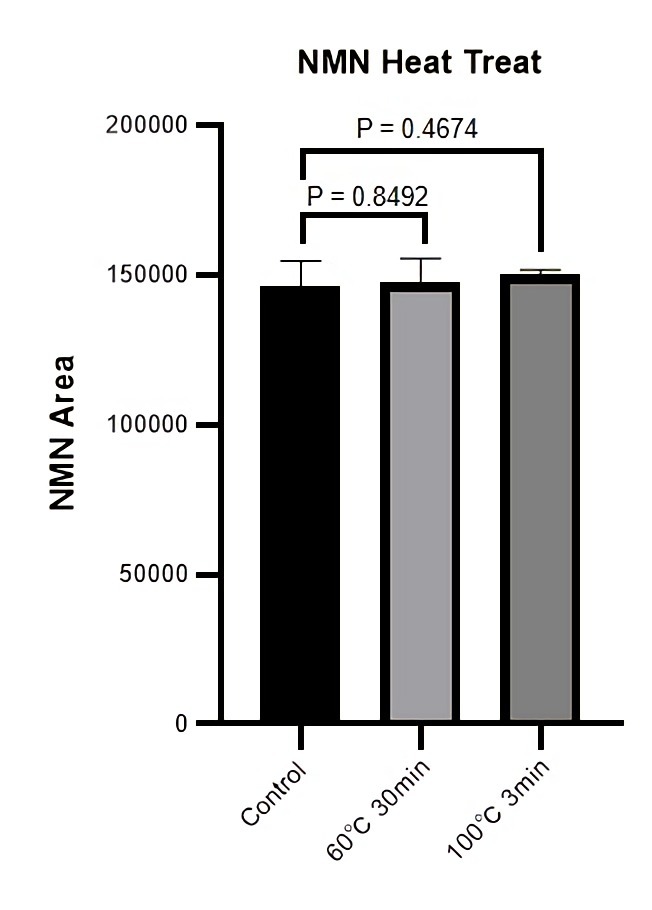

Supplement: Supplementary file 1 [file molecules-27-07054-s001.zip › S2.jpg]

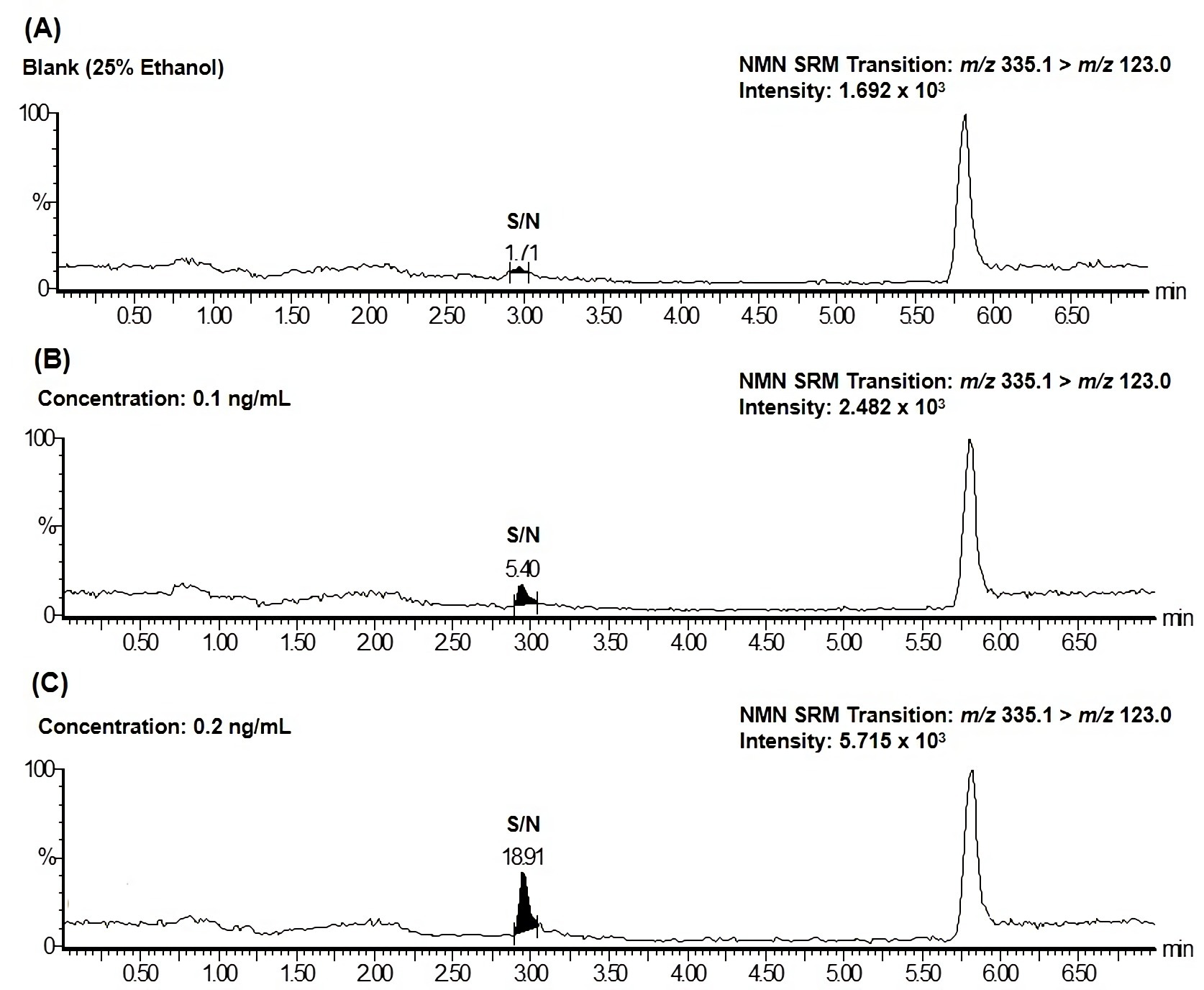

Supplement: Supplementary file 1 [file molecules-27-07054-s001.zip › S3.jpg]

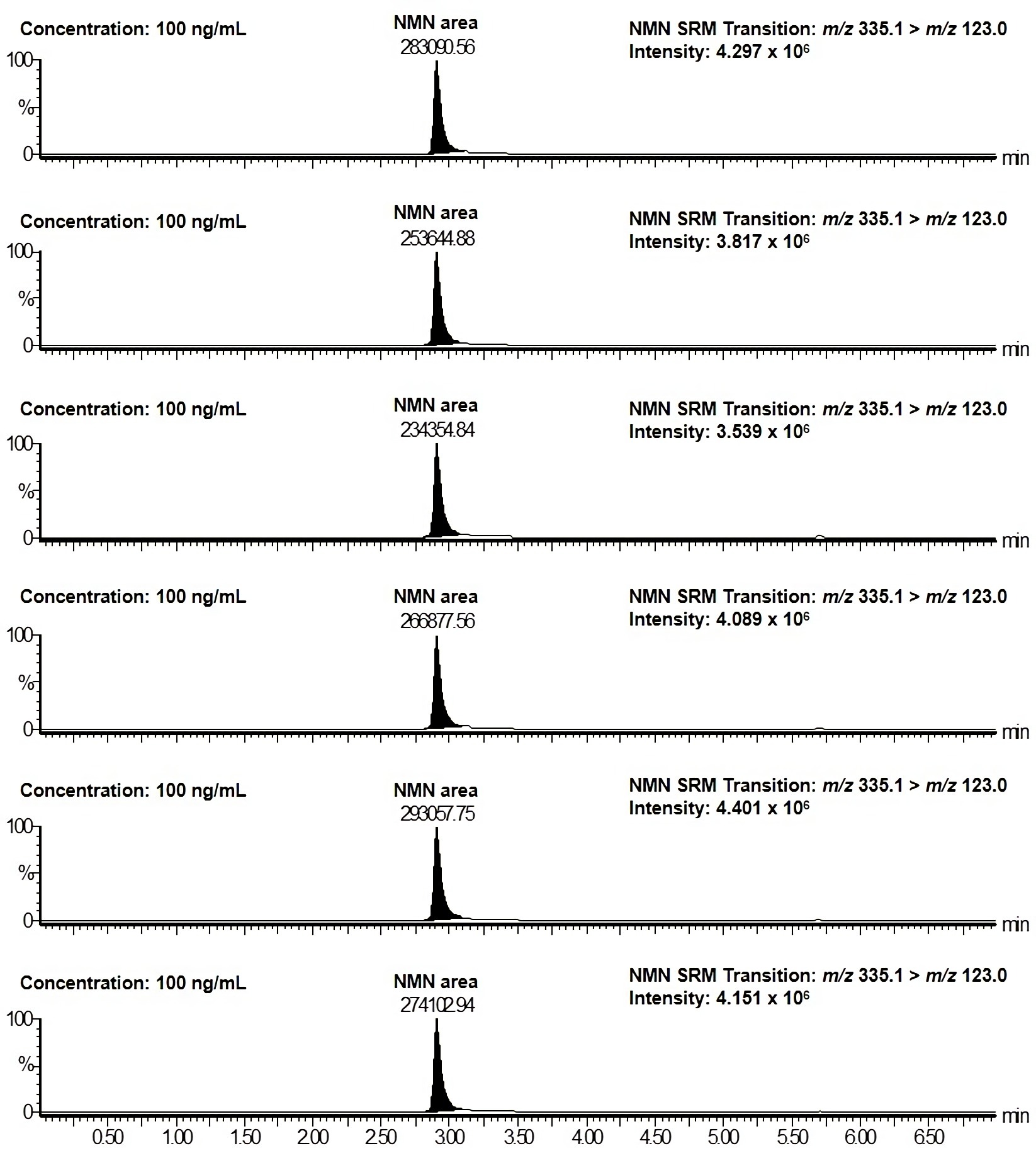

Supplement: Supplementary file 1 [file molecules-27-07054-s001.zip › S4 (2).jpg]

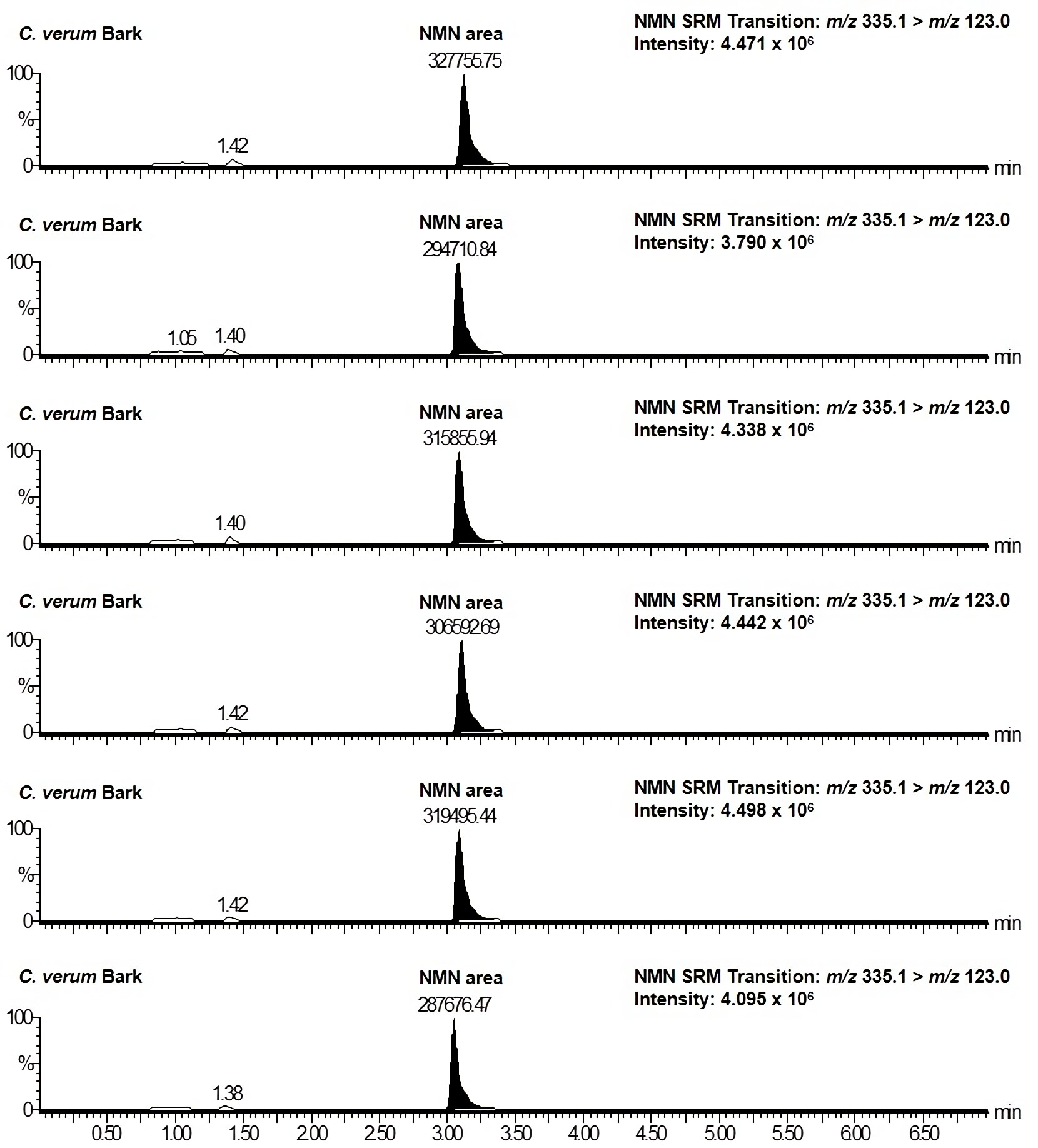

Supplement: Supplementary file 1 [file molecules-27-07054-s001.zip › S5 (1).jpg]
